# Minimally invasive treatment of complex collections: safety and efficacy of recombinant tissue plasminogen activator as an adjuvant to percutaneous drainage

**DOI:** 10.1590/0100-3984.2017.0086

**Published:** 2018

**Authors:** Priscila Mina Falsarella, Rafael Dahmer Rocha, Antonio Rahal Junior, Guilherme Falleiros Mendes, Rodrigo Gobbo Garcia

**Affiliations:** 1 MD, Physician in the Department of Interventional Radiology of the Hospital Israelita Albert Einstein, São Paulo, SP, Brazil.

**Keywords:** Abscess, Drainage, Fibrinolytic agents

## Abstract

**Objective:**

To analyze the efficacy of recombinant tissue plasminogen activator (r-TPA) injection in the evolution of percutaneous drainage of thick collections.

**Materials and Methods:**

This was a single-center study involving the retrospective analysis of hospitalized patients undergoing percutaneous drainage of thick (superficial or intracavitary) fluid collections, followed by injection of a fibrinolytic agent (r-TPA) into the affected space.

**Results:**

A total of 53 percutaneous drainage procedures, with r-TPA injection, were performed in 51 patients. Abdominal and pelvic collections were the most common, being seen in 38 (73%) of the procedures; in 35 (66%), the etiology of the collection was attributed to postoperative complications. A total of 61 catheters were used in order to drain the 53 collections. Of those 61 catheters, 52 (85%) were large (12-16 Fr) and 9 (15%) were small (4-10 Fr). The mean r-TPA dose was 5.7 mg/collection per day, and the mean time from r-TPA injection to drain removal was 7.7 days. Percutaneous drainage in combination with r-TPA injection was successful in 96% of the cases. None of the patients showed coagulation changes during the study period.

**Conclusion:**

The use of once-daily, low-dose r-TPA for up to three consecutive days, as an adjunct to percutaneous drainage of thick collections, with or without loculation, appears to be an effective technique.

## INTRODUCTION

Complex collections typically present a therapeutic challenge and can range from organized collections of blood with locoregional compressive symptoms to infectious processes that, in patients with various comorbidities, can result in unfavorable clinical outcomes such as sepsis and death^1^.

Complicated infection is a major cause of high morbidity and mortality, occurring most commonly in the abdominal cavity-in approximately 30% of cases^1^-and thoracic cavity-in 18-60%^2^. Among the main factors associated with a worse prognosis are worsening of severe underlying diseases, failure to determine the etiology of the collection, the use of inappropriate empirical antibiotic therapy, and infections caused by multidrug-resistant organisms. Early diagnosis, as well as the introduction of aggressive clinical and surgical therapies, are key to achieving favorable clinical outcomes^1^.

Minimally invasive percutaneous drainage guided by imaging, such as ultrasound and tomography, which are frequently used in conjunction^3^, is a well-established therapeutic option in the approach to collections at various sites, such as subcutaneous collections, as well as those in the intramuscular compartment, abdomen/pelvic cavity, and thorax^3-6^.

The advent of imaging equipment that provide better definition and various types of drains (to deal with superficial and deep collections of different viscosities) have resulted in an increase in the number of cases in which the minimally invasive technique is indicated, as well as improving outcomes. However, in thick, septated, encapsulated collections of blood containing debris, the minimally invasive approach provides less than satisfactory results^7-9^. In this context, the injection of fibrinolytic agents into the collection, in order to reduce the viscosity of the contents and facilitate the flow through the lumina of drainage tubes of different calibers^10,11^, represents a solution for the treatment of these less common cases in which the success of minimally invasive drainage is limited^8,9,12^, precluding the need for a more aggressive surgical approach, which is sometimes not applicable in patients who are more seriously ill.

The objective of this study was to analyze the safety and efficacy of recombinant tissue plasminogen activator (r-TPA) injection in the evolution of imaging-guided percutaneous drainage of thick fluid collections. We also describe the initial experience of the interventional radiology department of our hospital in the application of the technique.

## MATERIALS AND METHODS

This was a single-center study involving the retrospective analysis of patients submitted to percutaneous drainage of thick superficial or intracavitary fluid collections, followed by injection of a fibrinolytic agent, between April 2011 and May 2015. This study was approved by the medical ethics committee of the institution.

### Administration of r-TPA

The decision to use the fibrinolytic agent was made jointly between the interventional radiology team and the members of the clinical-surgical treatment team assigned to the patient.

After the appropriate positioning of the percutaneous drain had been verified by imaging, the fibrinolytic agent was injected through the lumen of the drain. The r-TPA was diluted in saline solution (ranging from 10 mL to 40 mL depending on the estimated initial volume of the collection) and instilled within the collection, with subsequent closure of the drain for 1 h. Subsequently, the drain was opened and the flow rate was measured, the measurement excluding the volume of solution administered. The treatment cycle consisted of administration of a daily dose of the saline-r-TPA solution for three consecutive days, unless the collection resolved spontaneously before that period. During administration of the fibrinolytic agent, the serum fibrinogen level was monitored and a coagulation test was performed.

## RESULTS

Percutaneous drainage of superficial or intracavitary collections, with r-TPA injection, was performed 53 times in 51 patients, two patients undergoing drainage of two different collections each. Of the 51 patients, 30 (58.8%) were women. The mean age of the patients was 55.6 years (range, 17-93 years).

Abdominal and pelvic collections were the most common (n = 38; 73%), followed by thoracic collections (n = 8; 15%) and collections in soft tissue (n = 7; 12%). The predominant cause of the collections was postoperative complications (n = 35; 66%), followed by pneumonia evolving to empyema (n = 8), liver abscess (n = 2), trauma (n = 2), complicated pancreatitis (n = 1), pyelonephritis (n = 1), renal cyst rupture (n = 1), loculated ascites associated with carcinomatosis (n = 1), and hematoma of the abdominal wall.

Drainage was performed with general anesthesia in 25 (49%) of the patients (n = 25), local anesthesia in 18 (35%), and local anesthesia combined with intravenous sedation in 8 (16%). Among the imaging methods used in order to guide the drainage, ultrasound alone was used in 27 (51%) of the procedures, computed tomography alone was used in 8 (15%), and the combination of the two methods was used in 18 (34%).

A total of 61 drains were used in order to access the 53 collections (45 were accessed with a single drain and 8 were accessed with two drains per collection). Of the 61 drains, 52 (85%) were large-caliber drains (12-16 Fr) and 9 (15%) were smaller-caliber drains (4-10 Fr).

The saline-r-TPA solution was administered after the correct positioning of the drain had been confirmed, being injected immediately after drainage in 28 (53%) of the cases, with a mean interval between the initial drainage and the r-TPA injection of 2.7 ± 4 days (range, 0-13 days).

The mean daily dose of r-TPA was 5.7 ± 3.0 mg/collection (range 2-10 mg), with a mean treatment duration of 2.6 days. The mean time between r-TPA injection and drain withdrawal was 7.7 days. There were no changes seen on the coagulation tests evaluated during the study.

**Figure 1:**
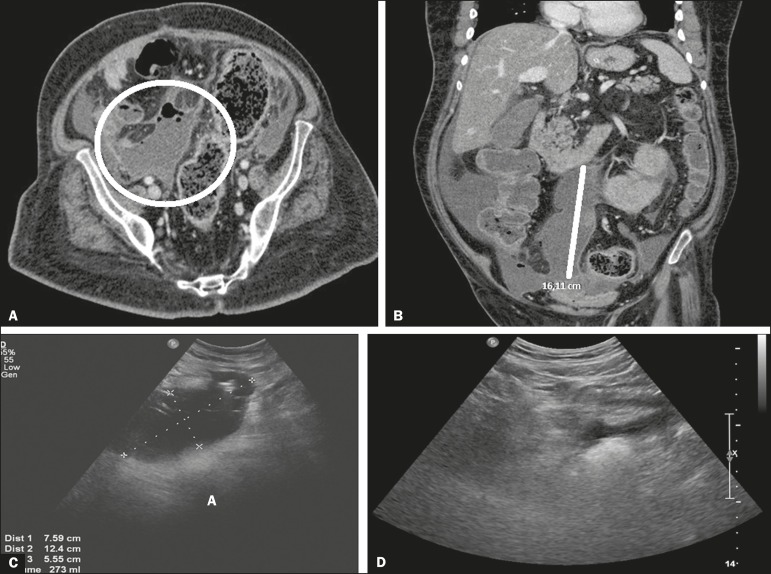
**A,B:** Computed tomography showing an intraperitoneal collection. **C:** Ultrasound six days after drainage of the collection, showing septa prior to r-TPA injection. **D:** Ultrasound three days after the end of the r-TPA cycle, showing resolution of the abdominal collection.

**Figure 2:**
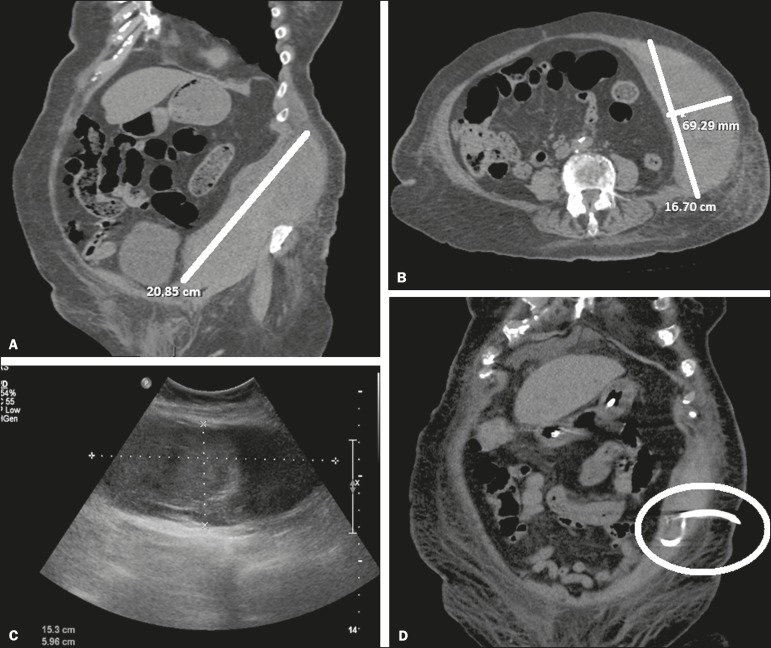
**A,B:** Computed tomography showing hematoma of the abdominal wall. **C:** Ultrasound after percutaneous drainage. **D:** Computed tomography after the end of the r-TPA cycle, showing resolution of the collection.

Among the collections treated with r-TPA injection, the overall success rate of percutaneous drainage in conjunction with the use of a fibrinolytic agent was 96%, successful drainage being achieved in 51 cases, with the following distribution (Figures 1 and 2): after one r-TPA cycle, in 45 cases (85%); after two r-TPA cycles, with resolution of the collection after the second cycle, in 1 (2%); and after placement of a second percutaneous drain in 5 (9%).

There were no cases of recurrence of the collection after drain removal. Percutaneous drainage was ineffective, making it necessary to drain the collection surgically, in only 2 cases (4%). In those cases, the percutaneous drain was removed during the surgical procedure. The only complication observed was bleeding in the collection bed, which occurred in one case, after the third day of the r-TPA cycle, in a collection secondary to early colorectal anastomosis fistula. The bleeding occurred in a patient with normal coagulation test results and was resolved through coil-spring embolization. No side effects or complications were observed during or after administration of the fibrinolytic agent in the other patients.

## DISCUSSION

Imaging-guided percutaneous drainage presents a high level of safety, with low rates of morbidity and mortality, whether performed with the Seldinger technique (using a guidewire) or with the tandem trocar technique (direct drainage of the collection, without the use of a guidewire), resulting in early recovery of patients in the post-procedure period^13^.

Despite the small number of studies on the injection of fibrinolytic agents into thick or loculated collections, some authors suggest that the injection of such agents increases the efficacy of percutaneous drainage of abscess, thus improving clinical outcomes^5^. Most such studies have involved the administration of urokinase or streptokinase^3,6,7,11,14-16^, although a few have used r-TPA^5,17^.

Our results demonstrate that the use of r-TPA in thick or septated collections, at a low single daily dose (mean, 5.7 mg) for up to three days, improved the evolution at different drainage sites, with a percutaneous drainage success rate of 96%. Froudarakis et al.^18^, in their sample of 20 patients, reported a 95% success rate in drainage of thoracic empyemas using a single daily dose of 25 mg of r-TPA for three days. Among the complications described, 25% of the patients presented pain during the administration of the fibrinolytic agent and 15% evolved to intrathoracic hemorrhage. Gervais et al.^17^ reported a success rate of 86% for drainage of intrathoracic collections with administration of low doses of r-TPA (4-6 mg), compared with the 73% success rate reported by Cheng et al.^19^ in patients with abdominal collections receiving r-TPA at doses of 2-4 mg. In both of those studies, the r-TPA was administered in three-day cycles, although it was injected twice daily. These results indicate that the technique is safer when lower doses (≤ 10 mg) are administered.

Lahorra et al.^20^ demonstrated the safety of the use of a fibrinolytic agent in intracavitary abscesses, reporting no changes in the coagulation test results. The patient in our sample who developed bleeding did not present systemic alterations on the coagulation tests. After a multidisciplinary discussion, it was decided that the bleeding could be attributed to the local effects of the fibrinolytic agent in the region of the collection, due to the fact that it is a friable area in the early postoperative period.

The major limitation of our study was its retrospective design, which precluded standardization of the r-TPA dose according to the volume of the collection.

## CONCLUSION

The use of a single low daily dose of r-TPA for up to three consecutive days, as a therapeutic adjunct to the percutaneous drainage of thick or loculated collections, proved to be a safe and effective technique that should be considered for inclusion in the minimally invasive arsenal for use in selected situations.
